# Bisphenol A affects vipergic nervous structures in the porcine urinary bladder trigone

**DOI:** 10.1038/s41598-021-91529-0

**Published:** 2021-06-09

**Authors:** Krystyna Makowska, Piotr Lech, Mariusz Majewski, Andrzej Rychlik, Slawomir Gonkowski

**Affiliations:** 1 Department of Clinical Diagnostics, Faculty of Veterinary Medicine, University of Warmia and Mazury in Olsztyn, Oczapowskiego 14, 10-957 Olsztyn, Poland; 2 Agri Plus Sp. Z O.O., Marcelinska Street 92, 60-324 Poznań, Poland; 3 Department of Human Physiology and Pathophysiology, School of Medicine, University of Warmia and Mazury in Olsztyn, Warszawska 30, 10-082 Olsztyn, Poland; 4 Department of Clinical Physiology, Faculty of Veterinary Medicine, University of Warmia and Mazury in Olsztyn, Oczapowskiego 13, 10-957 Olsztyn, Poland

**Keywords:** Neurophysiology, Cellular imaging, Environmental impact

## Abstract

Bisphenol A (BPA) is used in the production of plastics approved for contact with feed and food. Upon entering living organisms, BPA, as a potent endocrine disruptor, negatively affects various internal organs and regulatory systems, especially in young individuals. Although previous studies have described the neurotoxic effects of BPA on various tissues, it should be underlined that the putative influence of this substance on the chemical architecture of the urinary bladder intrinsic innervation has not yet been studied. One of the most important neuronal substances involved in the regulation of urinary bladder functions is vasoactive intestinal polypeptide (VIP), which primarily participates in the regulation of muscular activity and blood flow. Therefore, this study aimed to determine the influence of various doses of BPA on the distribution pattern of VIP-positive neural structures located in the wall of the porcine urinary bladder trigone using the double-immunofluorescence method. The obtained results show that BPA influence leads to an increase in the number of both neurons and nerve fibres containing VIP in the porcine urinary bladder trigone. This may indicate that VIP participates in adaptive processes of the urinary bladder evoked by BPA.

## Introduction

Bisphenol A (BPA), an organic synthetic chemical compound belonging to phenols, is widely used in the production of plastics since products made of BPA are characterised by strength and resistance to damage while being light and comfortable to use^1–3^. The widespread use of BPA and its capacity to flush out from plastics has contributed to the pollution of the air, soil and surface waters with this compound^1,4^. BPA may also enter humans and animals through the gastrointestinal tract, respiratory system or skin, wherein the gastrointestinal tract is the main route of penetration of this compound into the body^2^. Therefore, the greatest threat to living organisms is the presence of BPA in the food, bottles for drinking water, polyester resins for coating the inside of tin cans and dental materials^1,2^.

It is known that BPA exerts a variety of negative effects on many internal organs and regulatory systems related to its similarity to oestrogen and the possibility of stimulating oestrogen receptors. For this reason, BPA is included in the so-called “endocrine disruptor” group of chemicals^1,2^. Previous studies have reported that BPA causes pathological changes and disrupts the functions of the reproductive, endocrine, immunological, digestive, and cardiovascular systems^1,5–7^. BPA also greatly affects the central and peripheral nervous system^8–12^ by disrupting synaptogenesis and neurite growth, changing calcium metabolism and influencing the synthesis of neuronal active substances^13–16^.

Regarding the excretory system, the majority of previous studies have described the impact of BPA on kidneys, in which this endocrine disruptor causes disturbances in the development and reduction of efficiency^17^, resulting in a decrease in urine output and albuminuria^18^. Some studies have indicated a relationship between exposure to BPA and the risk of developing chronic kidney disease^19^. In contrast, the present knowledge about the possible influence of BPA on the urinary bladder is extremely scarce. Although previous studies have noted that exposure to BPA may result in urinary voiding dysfunction^20^ and contribute to inflammation and neoplasms^21^, there is not yet any data on the influence of this endocrine disruptor on the nervous structures in the wall of the urinary bladder.

Nervous structures of the urinary bladder include both neuronal cells grouped in intramural ganglia located mainly in the muscular and submucosal layers in the direct vicinity of the muscular layer. The most numerous intramural ganglia, which contain cell bodies of the post-synaptic parasympathetic neurons have been described in the urinary bladder of human, guinea pig and domestic pig, in which they are present especially in the urinary bladder trigone (part of the urinary bladder, limited by the ureteral openings and the internal opening of the urethra)^22–26^. Extrinsic innervation of the urinary bladder consist of three types of neurons: (1) sympathetic neurons located in the inferior hypogastric plexuses, inferior mesenteric ganglia and lumbar ganglia of the sympathetic trunk^27^; (2) parasympathetic neurons located in the sacral spinal cord, which processes form synapses with neurons in the intramural ganglia, as well as parasympathetic neurons located in the pelvic ganglia^28^ and (3) sensory neurons located in the thoracic, lumbar and sacral dorsal root ganglia (depending on species studied)^29^.

Studies conducted so far have shown that bladder intrinsic neurons may be divided into numerous neurochemical (= functional) subclasses based on the pattern of the synthesis and expression of neuronal active substances [for review, see^30^]. It should be stressed that despite the clear inter-species differences, the majority of intrinsic neurons within the urinary bladder wall contain vasoactive intestinal polypeptide (VIP), constituting a source of numerous VIP-ergic intramural nerves observed in all layers of the organ^24,25,31^. However, it should be noted that some of the VIP-positive fibres originate from extrinsic sources^32^. These observations strongly suggest that this polypeptide plays an important role in the regulation of urinary bladder functions.

VIP, one of the most important inhibitory neuronal factors found in the non-cholinergic and non-adrenergic (NANC) neurons, causes hyperpolarisation of both the vascular and non-vascular smooth muscle cells in many internal organs and therefore shows strong relaxant activity^33–35^. A similar impact of VIP on the smooth muscles has been described in the urinary bladder, although the degree of VIP-induced relaxation of muscles in this organ appears to be species-specific^32,36,37^. Furthermore, previous studies have also reported the participation of VIP in the conduction of sensory stimuli from the urinary bladder, as well as in adaptive and/or neuroprotective mechanisms, activated in pathological conditions of this organ^38–40^.

Therefore, the present study was aimed to investigate the influence of two different doses of BPA (the lower dose used in this study—0.05 mg/kg of body weight/day is regarded by regulations in some countries as a tolerable daily intake dose or reference dose of BPA completely safe for humans and animals) on the VIP-positive nervous structures located in the porcine urinary bladder trigone.

It should be pointed out that daily exposure of the living organisms on BPA in everyday life is difficult to exact establish, because it depends on numerous conditions, including environmental pollution, dwelling place, diet and even (in the case of people) chosen profession^1,41^. Moreover, BPA may affect organisms in a different way such the gastrointestinal tract, skin and respiratory system. In the case of farm animals the knowledge about the degree of daily exposure to BPA is extremely scanty. In turn, average human exposure is usually lower than dose fixed as a tolerable daily intake. Recently two methods of evaluation of exposure to BPA are used. According to the first of them-based on urinary excretion the average human exposure to BPA amounts to 2.53 µg/day (in regions with a high degree of environmental pollution 14.5 µg/day), and according to the second wastewater-based epidemiology method 513.73 µg/day^42^. On the other hand it is known that exposure of humans to BPA in some situations may be higher than 0.05 mg/kg b.w./day. Namely previous studies have reported that in regions with a high degree of environmental pollution the human exposure to BPA may reach above 11,550 µg/day, and in humans with reconstructions of molar teeth crowns the dose of BPA released from dental fillings may amount to even 30 mg/day^43,44^.

Moreover, due to the similarity in the bladder intramural innervation of humans and the domestic pig^22,24,25^, the results of this study may contribute to a better understanding of the impact of BPA on the human urinary tract.

## Results

**Figure 1:**
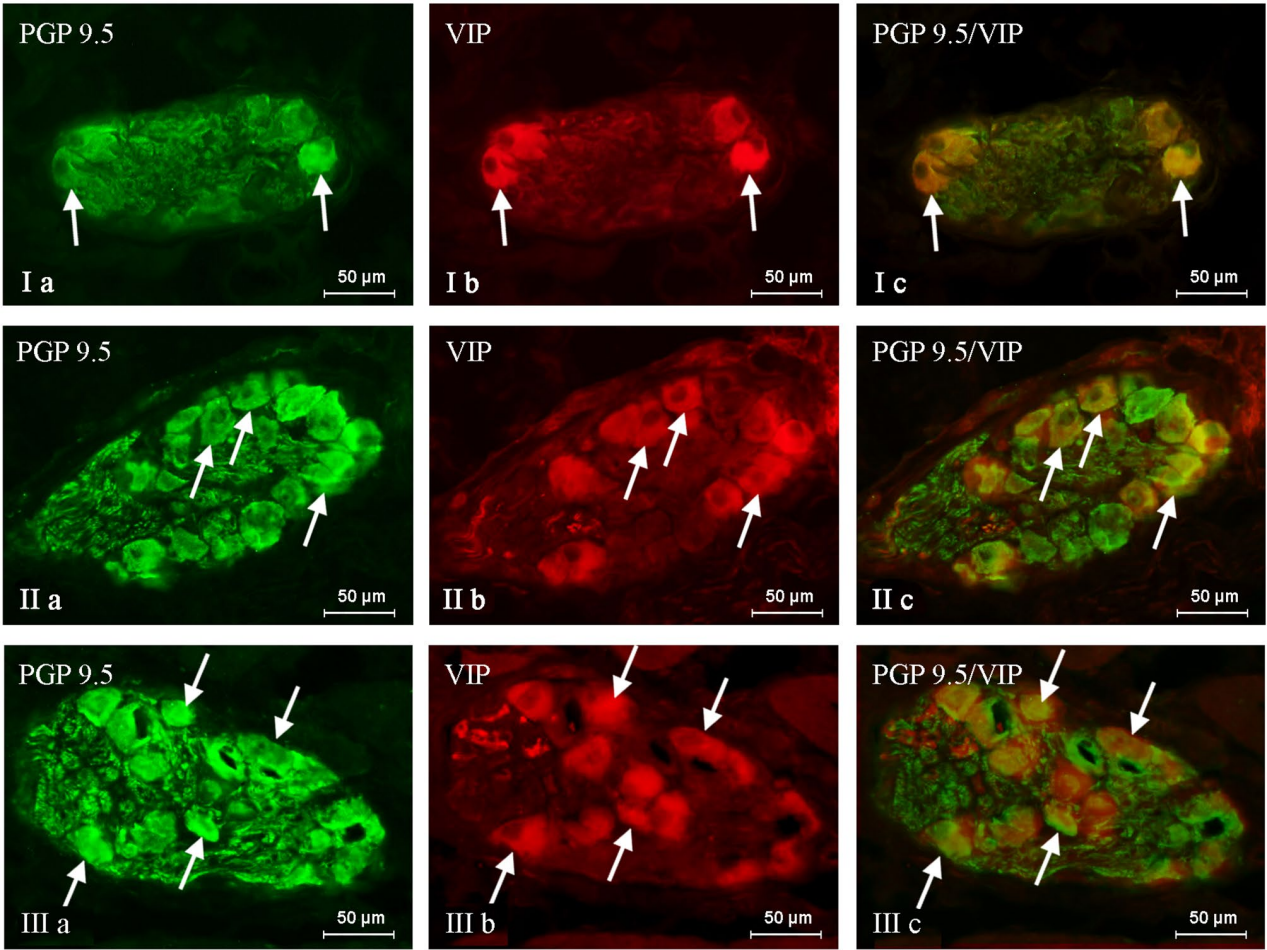
Neurons immunoreactive to protein gene product (PGP 9.5—green)—used here as a pan-neuronal marker and vasoactive intestinal polypeptide (VIP—red) in the intramural ganglia of the porcine urinary bladder trigone under physiological conditions—group C (I), under the influence of BPA in a dose of 0.05 mg/kg body weight/day—group I (II) and under the influence of BPA in a dose of 0.5 mg/kg b.w./day—group II (III). VIP—positive neurons are marked with arrows. The right column (**c**) was created by overlapping pictures (**a**) and (**b**).

**Figure 2:**
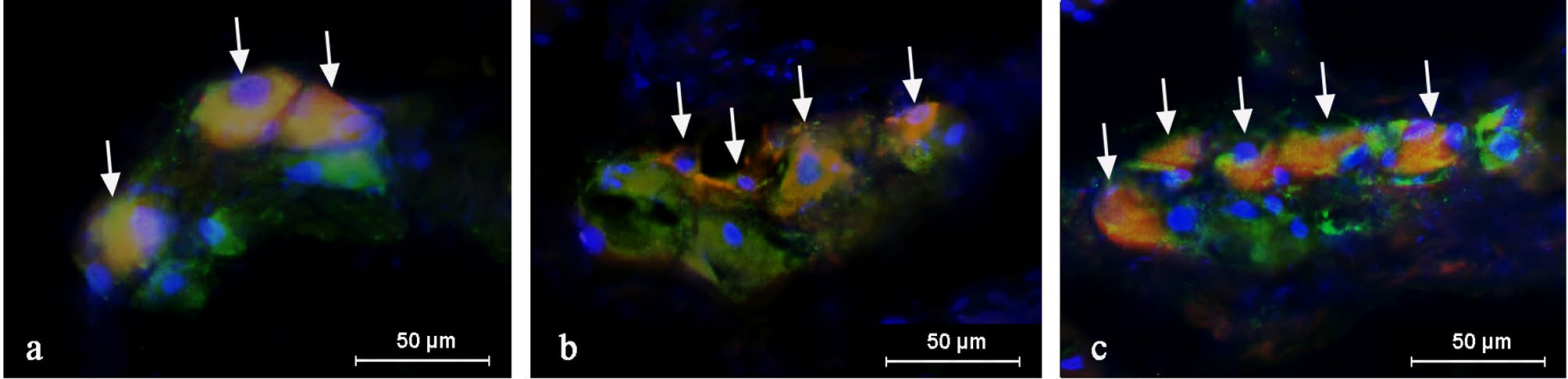
Intramural ganglia in the porcine urinary bladder in control animals (**a**) and animals treated with low (**b**) and high (**c**) doses of bisphenol A, labeled against pan-neuronal marker PGP 9.5 (green), vasoactive intestinal polypeptide—VIP (red) and a marker of cell nuclei-4′,6-diamidino-2-phenylindole (DAPI) (blue). VTP-positive neurons are indicated with arrows.

VIP-immunoreactive (VIP-IR) neuronal perikarya were found in intramural ganglia of the urinary bladder trigone in animals of all studied groups (Table 1, Figs. 1, 2). Under physiological conditions, the number of VIP-positive neurons was relatively high (Fig. 1) and amounted to 32.22 ± 0.57% of all intramural neurons labelled with pan-neuronal marker—protein gene product 9.5 (PGP 9.5) (Table 1). VIP-IR cells usually formed clusters of 3–4 perikarya that were irregularly dispersed in the ganglion. However, intrinsic ganglia devoid of visible VIP-positive neurons were also examined.

**Table 1:** The percentage of neurons immunoreactive to vasoactive intestinal polypeptide (VIP) in the wall of urinary bladder trigone (in relation to all PGP 9.5—positive cells) in the control group (C), the experimental group I (I) and experimental group II (II).

	C	I	II
Animal 1	31.14	39.68	42.83
Animal 2	33.33	42.03	45.58
Animal 3	32.00	44.73	47.91
Animal 4	30.88	43.54	44.97
Animal 5	33.73	43.43	43.48
Mean	32.22	42.68^a^	44.95^a^
SEM	0.57	0.86	0.89

The results were considered statistically significant at p < 0.05 and are marked with^a^.

**Figure 3:**
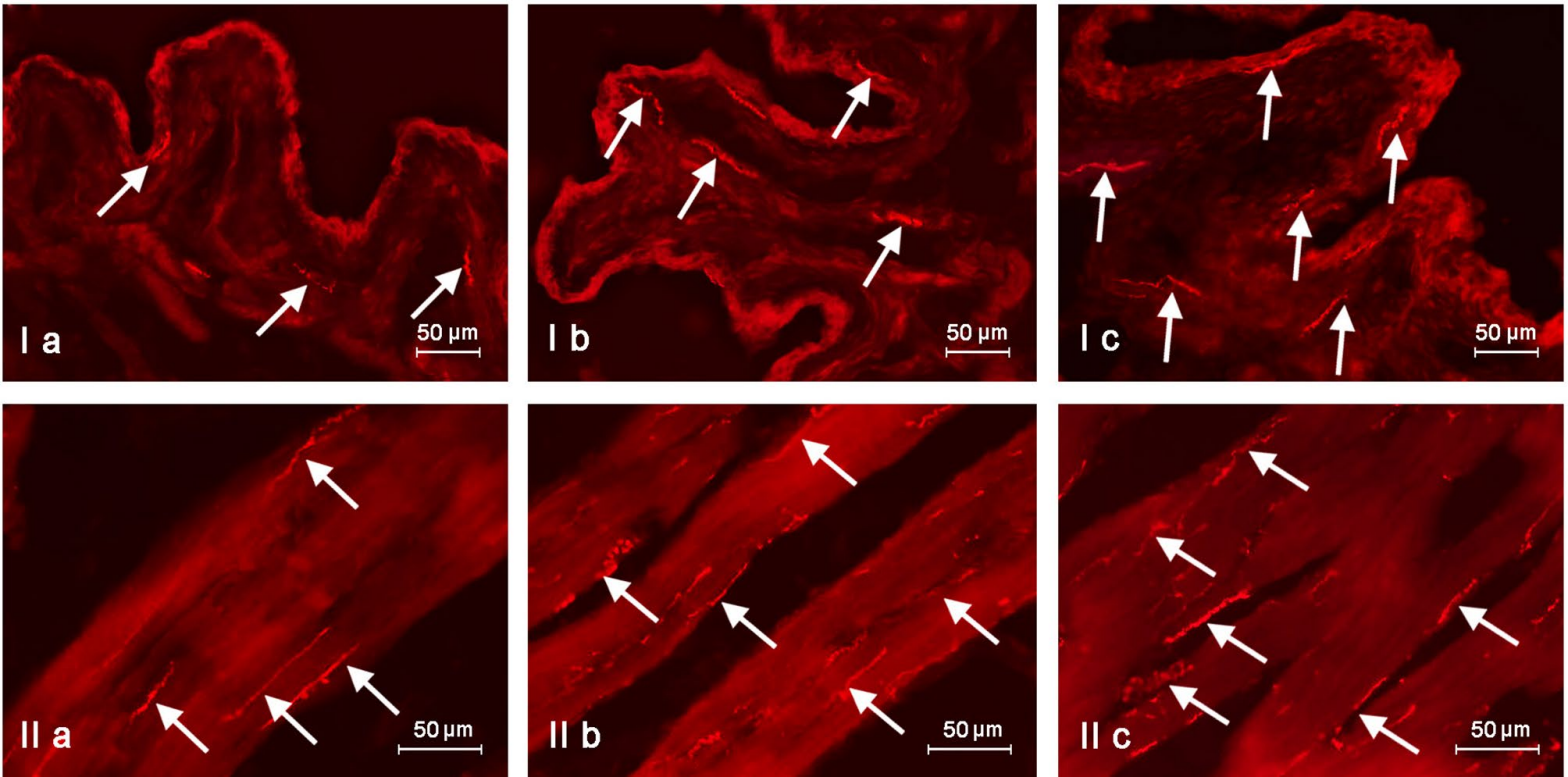
Nerves immunoreactive to vasoactive intestinal polypeptide (VIP) in the mucosal (I) and muscular (II) layers of the porcine urinary bladder trigone under physiological conditions (**a**), under the influence of BPA in a dose of 0.05 mg/kg b.w./day (**b**) and under the influence of BPA in a dose of 0.5 mg/kg b.w./day (**c**). VIP—positive nerves are marked with arrows.

The presence of VIP was also confirmed in nerve fibres located in the muscular and mucosal layers of the urinary bladder (Table 2; Fig. 3). In control animals, the average number of intramucosal VIP-IR nerve fibres amounted to 6.05 ± 0.36 fibres in the field of vision, while their average number in the muscular layer achieved 13.97 ± 0.12 per field of vision (Table 2). Clear morphological differences were observed between intramucosal and intramuscular VIP-containing nerves. While the majority of intramucosal VIP-positive nerves were rather delicate and thin, fibres located in the muscular layer were distinctly thicker.

**Table 2:** The average number of nerve fibers immunoreactive to vasoactive intestinal polypeptide (VIP) per observation field (0.1 mm^2^) in the wall of urinary bladder trigone in the control group (C), the experimental group I (I) and experimental group II (II).

	C	I	II
**Mucosal layer**			
Animal 1	5.55	8.70	13.30
Animal 2	5.45	10.02	12.85
Animal 3	7.45	9.45	14.15
Animal 4	6.00	11.05	13.67
Animal 5	5.80	12.68	15.45
Mean	6.05	10.38^a^	13.88^ab^
SEM	0.36	0.69	0.45
**Muscular layer**			
Animal 1	14.05	15.75	20.25
Animal 2	13.60	18.30	22.37
Animal 3	14.15	14.90	25.20
Animal 4	13.80	18.35	21.45
Animal 5	14.25	15.18	20.60
Mean	13.97	16.50^a^	21.97^ab^
SEM	0.12	0.76	0.89

The results were considered statistically significant at p < 0.05 and are marked with ^a^ (statistically significant differences between group I or II and group C) or ^b^(statistically significant differences between group I and II).

Although a statistically significant increase in the number of visible VIP-IR cells was observed after the exposure of animals to each dose of BPA, the severity of changes was dose-dependent. In group I (Fig. 1), the number of VIP—positive neurons amounted to 42.68 ± 0.86% of all nerve cells marked with PGP 9.5, while the number of neurons immunoreactive to VIP in animals of group II (Fig. 1) amounted to 44.95 ± 0.89% of all PGP 9.5-positive neuronal cells.

The distribution pattern of VIP-positive cells in the intramural ganglia in animals influenced by BPA was the same as in the control animals. However, contrary to animals of the control group, clusters of VIP-positive neurons (usually 4–5 cells) were more commonly observed and ganglia deprived of neurons-containing VIP were not observed in pigs treated with BPA.

Both doses of BPA used in this experiment caused an increase in the number of VIP-IR nerves supplying the mucosal and muscular layers of the urinary bladder wall (Table 2). After administration of the lower dose of BPA, the average number of nerve fibres containing VIP amounted to 10.38 ± 0.69 and 16.5 ± 0.76 8 per field of vision in the mucosal and muscular layer, respectively. In turn, in animals of group II, the average number of VIP-IR nerves per field of vision was 13.88 ± 0.45, and 21.97 ± 0.87 in the mucosal and muscular layer, respectively (Table 2). Moreover, the presence of BPA changed the light-microscopical morphology of VIP-IR nerves both in the mucosal and muscular layer, which was more visible after exposing the animals to the higher dose of BPA. VIP-containing nerves in animals treated with BPA were distinctly thicker than fibres observed in control animals. Moreover, during the study noticed that VIP-positive nerves in animals treated with BPA often formed thick bundles in the muscular layer, accompanying particular clusters of detrusor smooth muscle cells.

## Discussion

This investigation confirmed the presence of VIP in neural structures of the porcine bladder wall, both under physiological conditions (which fully corroborates previous observations in other species^45–47^) and after exposure of the animals to various doses of BPA.

Interestingly, results concerning the population size of neurons immunoreactive to VIP, observed in porcine intramural ganglia in this investigation differ from those described in previous experiments, where the lowest number of VIP-positive structures (about 5% of all intramural neurons) or even absence of such structures in the urinary bladder wall have been noted^24,25^. The reason for these discrepancies is not entirely clear. Although, these discrepancies may result from differences in the applied counting methods, the kind of antibodies used or the preparation of tissues for the immunofluorescence technique^24,25^, it is also possible that they may be sex-dependent or reflect the interracial variations in the distribution of VIP in urinary bladder wall structures (which is contrary to this experiment but in the line with previous studies conducted on the pigs of Polish Great White breed). However, it cannot be excluded that the most likely explanation of the observed differences was the influences of unspecified environmental factors, such as the type of diet, the composition of feed or even characteristics of microorganisms of a given environment. Thus, further studies are needed to unveil the detailed reasons leading to different patterns of neurochemical characteristics of urinary bladder intrinsic neurons.

The relatively high number of VIP-positive intramural neurons, as well as the density of VIP-ergic nerve fibres located in the muscular and mucosal layers, noted in the present study, suggest that this substance plays an important and, most probably, multidirectional role in the neural regulation of the urinary bladder functions. However, it should be emphasised that the exact roles of VIP in the urinary bladder wall or even functions of the intramural ganglia, neurons, which may contain active substances typical for the parasympathetic and sympathetic nervous systems^24,25,31,43,44,48,49^ have not been completely clarified to date. Although VIP is commonly considered to be an important factor with hyperpolarizing properties, showing an inhibitory effect on the activity of both the vascular and non-vascular smooth muscles^33–35^, previous experiments have shown that its roles in the urinary bladder wall are not so explicit and clearly depend on the studied species^32^. In humans and pigs, VIP has a strong relaxant effect on the detrusor muscle, while this activity is much weaker in rodents^32,36,37^. In addition, irrespective of its origin (intrinsic bladder neurons or extrinsic perikarya of the parasympathetic (i.e., pelvic), sympathetic (para- as well as prevertebral) or dorsal root ganglia^39,50,51^, VIP is an important factor influencing the activity of intramural neurons in the bladder wall, as well as an agent involved not only in the regulation of blood flow but also in the modulation of immune processes^31,52–54^.

During the present experiment, it has unequivocally been shown that both doses of BPA, administered orally for a relatively short period of time, significantly affect the number of VIP-positive neuronal structures in the wall of the urinary bladder. However, it should be emphasised that due to the lack of studies directly addressing this issue, as well as the well-known multidirectional effects of BPA on a living organism^1,2^, the exact reasons and mechanisms of the observed changes cannot be clearly defined at the moment. Since they may be a reflection of neurotoxic, proinflammatory or neurodegenerative reactions induced in the target cells by exposure to BPA (see below), further studies on the exact etiopathogenesis of observed changes are necessary to verify the above-mentioned hypotheses.

Thus, the fluctuations in the number of VIP-IR perikarya and nerve fibres observed in this study may be a result of the neurotoxic activity of BPA. Previous studies have clearly shown that BPA not only impairs synaptogenesis but also already existing synapses (leading to the classical case of synaptic stripping). It has also been demonstrated that this compound seriously distorts the development of dendritic projections and inhibits the growth of neuronal axons^9,13,14,55^. The direct effect of BPA on neuronal cells and nerve fibres is probably associated with the injury of calcium channels in neurolemma and changes in the concentration of calcium ions inside the neuronal cells^56,57^. Furthermore, as implied by other studies, BPA alters the level of certain active substances, including calcium-calmodulin-activated protein kinase II, synaptophysin, galanin, as well as substance P in both the central and peripheral nervous systems^11,15,16^.

It is worth mentioning that VIP is known as one of the most potent neuroprotective factors in the central and peripheral nervous systems^58,59^. Previous publications have shown that this substance enhances the survival rate of nerve cells subjected to various pathological factors^58^. VIP has also been found to be involved in early embryonic development^60^, differentiation of embryonic stem cells^61^ and control of regulatory processes related to mitosis, differentiation and survival of neuroblasts in cell cultures^62^. VIP also inhibits the development of neurodegenerative diseases^63^. The neuroprotective activity of VIP is most likely connected with the effect of this peptide on glial cells, which, in turn, leads to the stimulation of these cells to synthesize and release anti-inflammatory cytokines^64^.

Although the exact neurodegenerative effect of BPA on the neuronal structures supplying the urinary bladder has not yet been confirmed, previous observations indicating BPA-induced urinary voiding disruption^20^, strongly suggest that such phenomenon may also be observed in this organ, similar to other parts of the nervous system^65,66^. Thus, considering the neurotoxic effects of BPA and VIP’s role as a key neuroprotective factor for autonomic neurons, the observed fluctuations in the expression pattern of VIP—were most probably of a compensatory nature. They may result from the increase in the VIP synthesis (as a neuroprotective factor, which prevents the toxic activity of BPA) in neuronal structures, which in physiological conditions do not show the presence of this substance and were aimed at maintaining homeostasis, providing affected neurons with abilities to perform their functions in the environment changed by BPA. This hypothesis is even more probable as a similar situation has previously been noted in the nervous structures supplying other internal organ not only under the impact of BPA, but also in other pathological states^67–69^.

The increase in the number of VIP-IR nervous structures noted in the present investigation may also be connected with inflammatory processes. Although BPA is a potent pro-inflammatory agent^70^, VIP exerts strong and well-known anti-inflammatory effects^71^. In particular, it is known that, apart from VIP-induced stimulatory effect on glial cells, leading to the increase in the secretion of an array of interleukins (ILs) (including IL-1α and β, IL-3 and IL-6), this peptide may inhibit the activity of macrophages, resulting in a decrease in the production of pro-inflammatory cytokines^72–74^. Moreover, VIP reduces the severity of the stimulation of antigen-specific CD4+ T cells and promotes the response of type 2 helper T cells^75^. Therefore, it cannot be ruled out that the changes in the number of VIP-IR structures noted in this experiment are the first manifestations of subclinical inflammatory processes. This hypothesis is even more likely, although the experimental animals showed no clinical signs of cystitis, since both the increase in the number of nervous structures-containing VIP in various internal organs, as well as strict correlations between the nervous and immune systems, are well-known from previous studies^76–78^.

Another “trigger” of the observed changes may be the direct impact of the studied endocrine disruptor on muscular activity. The latter supposition results from the fact that although BPA shows a relaxant impact on the smooth muscles^79^. On the other hand VIP is regarded as a potent neuronal factor inhibiting the activity of such muscles in various internal organs^35^. Therefore, the synthesis of VIP in neuronal cells (which in physiological conditions do not produce VIP), as well as enhancing the transport of this substance from cell bodies to nerve endings resulting in the increase in the number of nervous structures containing VIP in the urinary bladder wall may be, at least in part, connected with the relaxant activity of BPA.

To sum up, it should be emphasised that the results obtained in this experiment indicate that even low doses of BPA administered for a relatively short period are not devoid of impact on a living organism, as they cause clear changes in the number of VIP-IR nerve structures located in the urinary bladder wall. This research is the first study on the influence of BPA on the innervation of the urinary bladder and, due to the multidirectional effects of BPA on the body and the diverse roles of VIP in the nervous system, it is very difficult to accurately determine the causes and mechanisms of the observed changes. Nevertheless, it can be assumed that fluctuations in the expression of VIP in the neural structures of the urinary bladder wall result from neurotoxic and/or proinflammatory properties of BPA and changes in the population of VIP-positive nervous structures may be the first manifestation of intoxication with BPA, even at relatively low doses.

## Materials and methods

The present investigation involved 15 immature Pietrain × Duroc mixed-breed porcine females (8 weeks old, approx. 20 kg of body weight) kept under standard experimental conditions. The pigs were fed twice a day with a properly composed commercial fodder and they were provided access to water ad libitum. All experimental protocols were approved by the Local Ethical Committee for Animal Experiments in Olsztyn, the University of Warmia and Mazury in Olsztyn, according to the Act on the Protection of Animals for Scientific or Educational Purposes of 15 January 2015 (Official Gazette 2015, No. 266), applicable in the Republic of Poland (based on the consent No. 28/2013/N of 22 May 2013). All of the applied methods were applied in accordance with the relevant guidelines and European and Polish regulations. Moreover, the study was carried out in compliance with the ARRIVE guidelines. In this study, after a few days of adaptation, animals were divided into three five-animal groups (two experimental and one control). For the next 28 days, pigs from each group received gelatin capsules before the morning feeding. The animals from the control group (Group C) received empty gelatin capsules and animals from the experimental groups were treated with capsules containing BPA. Capsules for the experimental group I (Group I) were filled with BPA in a dose of 0.05 mg/kg of body weight (b.w.)/day and capsules for experimental group II (Group II) were filled with BPA in a dose of 0.5 mg/kg b.w./day.

After 28 days of BPA administration, the animals were premedicated with azaperone (Stresnil, Janssen, Belgium, 0.8 mg/kg b.w., administered intramuscularly) and after about 30 min, euthanised with an overdose of sodium thiopental (Thiopental, Sandoz, Kundl, Austria, administered intravenously as a bolus injection). Immediately after the death of the animals, the urinary bladder trigones were collected and put into a 4% paraformaldehyde buffer (pH 7.4) for about 40 min (at room temperature). Afterwards, tissues were rinsed with a phosphate buffer (0.1 M, pH 7.4, temp. 4 °C) for 72 h with a buffer change every 24 h. Next, they were put into an 18%-buffered sucrose solution and kept at 4 °C for at least 3 weeks.

The properly prepared urinary bladder trigone fragments were then frozen at − 22 °C and cut perpendicular to the organ lumen into 14 µm slices with a cryostat (HM 525, Microtom International, Germany). The slices were placed on basic microscope slides and stored at − 22 °C until the next step.

Sections of the urinary bladder wall were subjected to standard double immunofluorescence labelling, as previously described in detail by Makowska^67^. Briefly, after removal from the freezer and 1-h drying, sections were covered by a drop of “blocking” solution (10% normal goat serum, 0.1% bovine serum albumin, 0.01% NaN3, 0.25% Triton X-100 and 0.05% thimerosal in PBS) for another one hour. After a repetitive washing in PBS, a mixture of primary antibodies was then imposed on the tissue fragments. This step was performed in the humid chamber and lasted overnight. In this study, a mixture of the two following antibodies was used: antibody directed towards PGP 9.5—used here as a pan-neuronal marker (Biogenesis, UK, code 7863‐2004, working dilution 1:2000) and antiserum directed towards VIP (Cappel, Aurora, OH, USA, code 11428, working dilution 1:5000). This labelling step was completed by repetitive rinsing in PBS. Sections of the urinary bladder trigone were then incubated with a mixture of two species-specific secondary antibodies conjugated with two various fluorochromes: Alexa Fluor 488 and Alexa Fluor 546 (both reagents from Invitrogen, Carlsbad, CA, USA, working dilution 1:1000) for 1 h. At the end of labelling the microscopic slides with tissue sections were treated with buffered glycerol and covered with the coverslips. After every step of the immunofluorescent labelling method, the urinary bladder slices were rinsed in PBS (3 × 10 min). Some urinary bladder slices were additionally subjected to routine labelling with a marker of cell nuclei—4′,6-diamidino-2-phenylindole (DAPI—Merck, Warsaw, Poland, code 10236276001).

Standard controls, i.e. pre-absorption of the antisera with appropriate antigen, omission and replacement of primary antisera by non-immune sera were performed to test the antibodies and specificity of the method.

To determine the percentage of neuronal cells immunoreactive to VIP, located in the intramural ganglia of the urinary bladder trigone wall, at least 500 PGP-9.5-positive cell bodies were examined for the presence of VIP. Double-labelled cells (only neurons with a clearly visible nucleus) were evaluated under an Olympus BX51 microscope (Olympus, Tokyo, Japan) equipped with epi-fluorescence and appropriate filter sets. The results were pooled and presented as mean ± SEM. To prevent the double counting of neurons, the sections admitted to the experiment were located at least 50 μm apart from each other.

Moreover, the evaluation of the density of VIP-IR nerves in the muscular and mucosal layers was based on counting nerve fibre numbers per field of vision under a microscope (0.1 mm^2^). The total number of nerve fibres in four fragments of the urinary bladder per animal (in five fields per section) was determined and the obtained data were pooled and presented as mean ± SEM.

All images were captured by a digital camera connected to a PC. The statistical analysis of the obtained results was performed using the one-way Anova test with post hoc analysis (Duncan’s test) using the Statistica 13.3 software package (StatSoft Inc., Tulsa, OK, USA). The data were normally distributed and had equal variance. The results were considered statistically significant at p < 0.05.
